# Hybrid *de novo* genome assembly data and comparative genomics of *Fusarium chlamydosporum* isolated from infected blackberry fields

**DOI:** 10.1016/j.dib.2025.111854

**Published:** 2025-07-09

**Authors:** Anton Pashkov, José Pedro Martínez-Hernández, Alfredo Herrera-Estrella, Pablo Cruz-Morales, Nelly Selem-Mojica, José Manuel Villalobos-Escobedo

**Affiliations:** a Escuela Nacional de Estudios Superiores unidad Morelia, Universidad Nacional Autónoma de México, Morelia, Mexico; b Unidad de Genómica Avanzada, Cinvestav, 36824 Irapuato, Guanajuato. Mexico; c Yeast Natural Products, The Novo Nordisk Foundation Center for Biosustainability, Technical University of Denmark, Denmark; d Tecnológico de Monterrey, Institute for Obesity Research, Ave. Eugenio Garza Sada 2501, Monterrey, N.L., 64849, Mexico; e Centro de Ciencias Matemáticas, Universidad Nacional Autónoma de México, Morelia, Mexico; f The LatAmBio Initiative, Irapuato, Guanajuato, Mexico

**Keywords:** Fusarium, Pathogenic strain, Genomics, Blackberry infection, Phylogenomics

## Abstract

Here, we present a dataset of ITS and β-tubulin sequences, along with a phylogenetic analysis using these molecular markers, for six putative *Fusarium* strains isolated from blackberry field soil. Additionally, we report the sequencing and hybrid assembly of one of these strains, identified as *Fusarium chlamydosporum*. Whole-genome sequencing of this strain was conducted using Illumina NovaSeq 6000 and Oxford Nanopore MinION technologies, resulting in a high-quality hybrid assembly of approximately 37 Mb across 23 contigs, with over 99 % completeness. Phylogenomic analysis, incorporating 88 *Fusarium* genomes, confidently placed this strain within the *F. chlamydosporum* clade. This assembly is now the NCBI reference genome for *F. chlamydosporum*, providing a valuable resource for future genomic and evolutionary studies of this species.

Specifications Table

**Table:** 

Subject	Biology
Specific subject area	Bioinformatics, Genomics
Type of data	Raw, Filtered, Figures, table, and Processed.
Data collection	Phylogenetic analysis: ITS and beta-tubulin regions were amplified by PCR and sequenced using Sanger technology to determine the strain's species. Phylogenetic analysis was conducted using MEGA11 software. Genomic DNA: Quick-DNA Fungal/Bacterial Microprep Kit (Zymo Research Corporation). Assembly: Genome assembly was performed with MaSURCA 4.1.1. Quality and completeness of the *F. chlamydosporum* genome were assessed with BUSCO 5.6.1.
Data source location	Data source location Institution: Tecnológico de Monterrey, Institute for Obesity Research; Centro de Ciencias Matemáticas, Universidad Nacional Autónoma de México; Unidad de Genómica Avanzada, Cinvestav. City/Province/Country: Monterrey/Nuevo León/México;Morelia/Michoacan/México; Irapuato/Guanajuato/México.
Data accessibility	1. PCR sequences Repository name: Zenodo Direct URL to data: https://doi.org/10.5281/zenodo.14715073 2. Raw sequences Repository name: NCBI SRA Data identification number: SRA: SRR32193854 (Illumina) and SRR32193853 (Oxford Nanopore) 3. Genome sequences Repository name: BioProject Data identification number: PRJNA1213090 Direct URL to data: https://www.ncbi.nlm.nih.gov/bioproject/PRJNA1213090/ Repository name: NCBI Genome Data identification number: GCA_047716405.1 Direct URL to data: https://www.ncbi.nlm.nih.gov/datasets/genome/GCA_047716405.1/ Repository name: NCBI Nucleotide Data identification number: JBLAUX000000000.1 Direct URL to data: https://www.ncbi.nlm.nih.gov/nuccore/JBLAUX000000000.1
Related research article	None

## 1 Value of the Data

• We present a new high-quality genome that represents an entire phylogenetic clade within the *Fusarium* genus, which is highly significant due to its potential plant pathogenicity.
• This comprehensive genomic assembly offers researchers a valuable tool for exploring the sequence space and identifying causal elements linked to the pathogenicity and emergence of *Fusarium chlamydosporum*.
• This new genome, being substantially more complete than the reference genome for this species, holds great potential for GWAS analyses. Facilitating future access and exploration by other researchers, the genome is now deposited in NCBI and is available under the GenBank accession GCA_047716405.1 and it is now the reference genome for the species *Fusarium chlamydosporum*.

## 2 Background

Fungi of the genus *Fusarium* are plant pathogens that cause diseases in many economically important crops, including cereals and vegetables [1]. *Fusarium chlamydosporum* is a soilborne pathogen, that is a facultative plant parasite [1,2] and a potential human pathogen [3]. In 2018, a high-yield blackberry production field in Los Reyes, Michoacán, Mexico, experienced a severe Fusarium infection, leading to significant economic losses for the producers. We collected samples from the soil and identified the microorganisms prevalent in the infection. We were also able to isolate and identify the strain IraGTOF6, which belongs to the species *F. chlamydosporum.* Phylogenomic analysis places our isolate IraGTOF6 in the *F. chlamydosporum* complex clade—a large and distinct group separate from the *F. oxysporum* clade. This suggests that the pathogenic properties of this strain may arise from unique genomic features that differ from those of more commonly studied pathogens. Only one genome of *F. chlamydosporum* has been reported and corresponds to strain NRRL 13,444.

## 3 Data Description

ITS and β-tubulin markers were amplified from DNA of the isolated fungus and sequenced, and the sequences were used to construct a phylogenetic tree, available in Zenodo (https://doi.org/10.5281/zenodo.14715073). These phylogenetic trees indicated that the IraGTOF6 strain most likely belongs to the species *F. chlamydosporum*. Additionally, we present a new genome assembly using long-read sequencing, which has been deposited in NCBI under the BioProject: PRJNA1213090. A phylogenomic analysis including all sequenced genomes of the *Fusarium* genus was also conducted and discussed.

### 3.1 ITS phylogeny clustered IraGTOF6 with *F. chlamydosporoum*

**Fig. 1:**
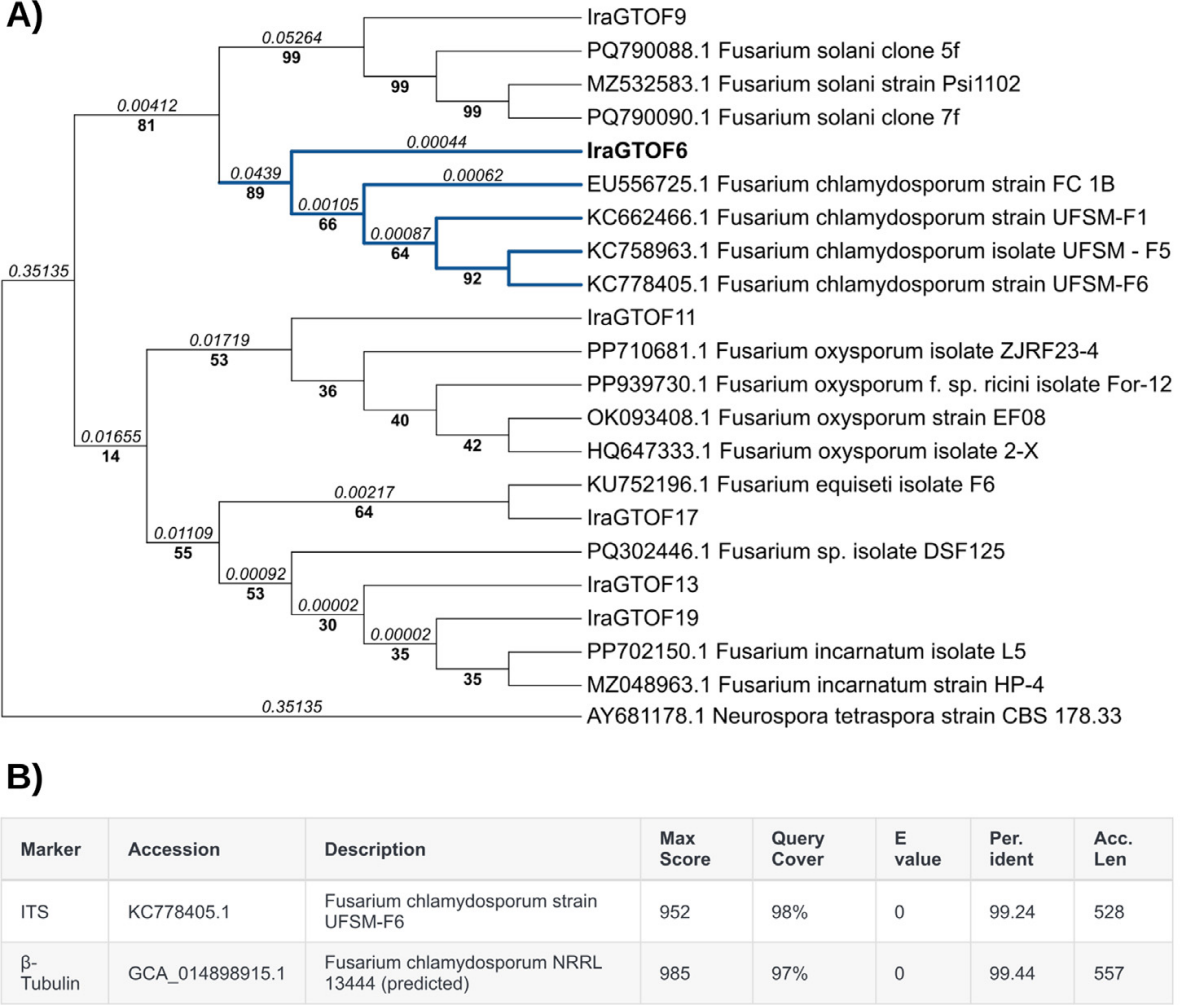
IraGTOF6 most likely belongs to *Fusarium chlamydosporum* based on phylogenetic analysis and BLAST results. A) A phylogenetic tree was constructed using the ITS sequences (∼500 bp) from six field isolates (strain names “IraGTO”) associated with fusariosis and 17 ITS sequences extracted from the NCBI database as references. The tree was built using the Neighbor-Joining model, and 1000 bootstrap replicates were used to validate the branch support. B) Table showing the BLAST results for the ITS and β-tubulin markers of the IraGTOF6 isolate. *Per. Ident*: percentage of identity; *Acc. Len*: alignment length.

The analysis of the ITS sequences obtained from the DNA of fungi isolated from soil samples revealed that *Multiple species* of *Fusarium* were involved in the infection (Fig. 1A). Strain IraGTOF6 stands out because it is not an *F. oxysporum*, the most common blackberry pathogen [4]. IraGTOF6 by ITS sequence is closely related to *F. chlamydosporum* NRRL 13,444. To further support this taxonomic classification, we performed a phylogenetic analysis using the β-tubulin gene, which confirmed that IraGTOF6 belongs to the species complex of *F. chlamydosporum* (Fig. 1B, Fig. S1).

**Fig. 2:**
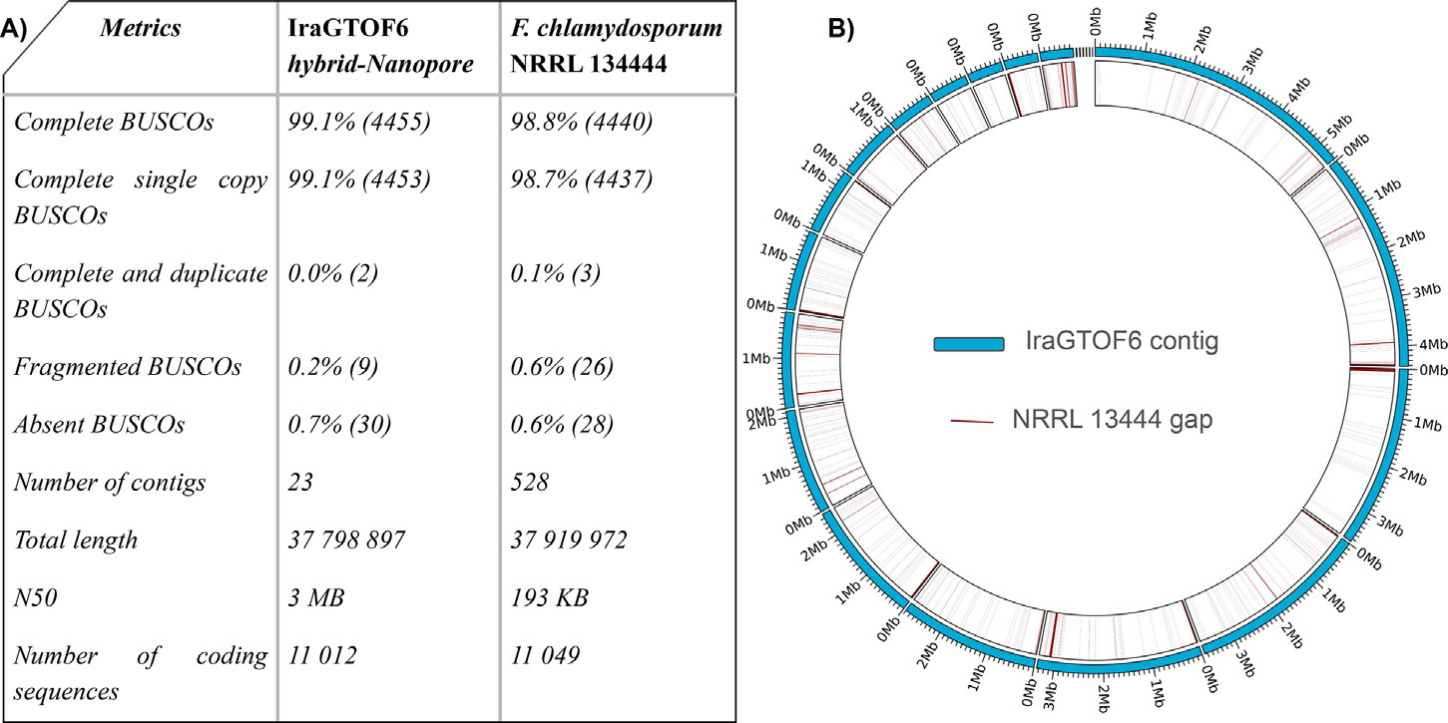
The hybrid assembly of IraGTOF6 shows significant improvements in contiguity and completeness over NRRL13444. A) Overview of whole-genome shotgun sequencing project descriptions and global genome assembly statistics utilizing Illumina and Oxford Nanopore technologies compared to the previously reported *F. chlamydosporum* genome. The Hypocreales lineage, from which BUSCOs were used to assess genome completeness, comprises 4494 BUSCOs. As such, the percentage values represent the proportion of BUSCOs within each category, while the values in parentheses indicate the total counts for the respective categories. B) Circos plot highlighting the fragmented nature of NRRL13444, the previous reference genome for *F. chlamydosporum*. Contigs in IraGTOF6 are represented in blue in the exterior circle, while red lines represent gaps in the NRRL13444 genome.

We performed a hybrid genome assembly of IraGTOF6 by integrating the data from Illumina and Oxford Nanopore platforms. Key genome assembly metrics were then compared between the hybrid Illumina-Nanopore assembly and the current *F. chlamydosporum* reference genome (Fig. 2A). The assembly completeness was determined by an analysis based on BUSCOs from the Hypocreales order, which is the closest taxonomic level to *F. chlamydosporum* available in BUSCO. The hybrid assembly achieved a high completeness, at 99.1 %. The genome consists of 11,365 genes, of which 11,012 are protein-coding. Repetitive sequences comprise around 3 % of the genome, most of which are low complexity regions and simple repeats and include a total of 444 transposable elements.

In comparison, the *F. chlamydosporum* NRRL13444 strain genome exhibited a completeness of 98.8 %. The hybrid assembly (37,798,897 bp) and the NRRL13444 genome (37,919,972 bp) are consistent in size. Still, our assembly significantly reduces genome fragmentation from 23 contigs to the highly fragmented NRRL13444 genome having 528 contigs (Fig. 2B). Our assembly comprises 17 contigs bigger than 600Kb and six smaller ones. This reflects superior contiguity in the hybrid assembly.

### 3.2 Phylogenomics supports IraGTOF6 as *F. chlamydosporum* within a specific *Fusarium* clade

**Fig. 3:**
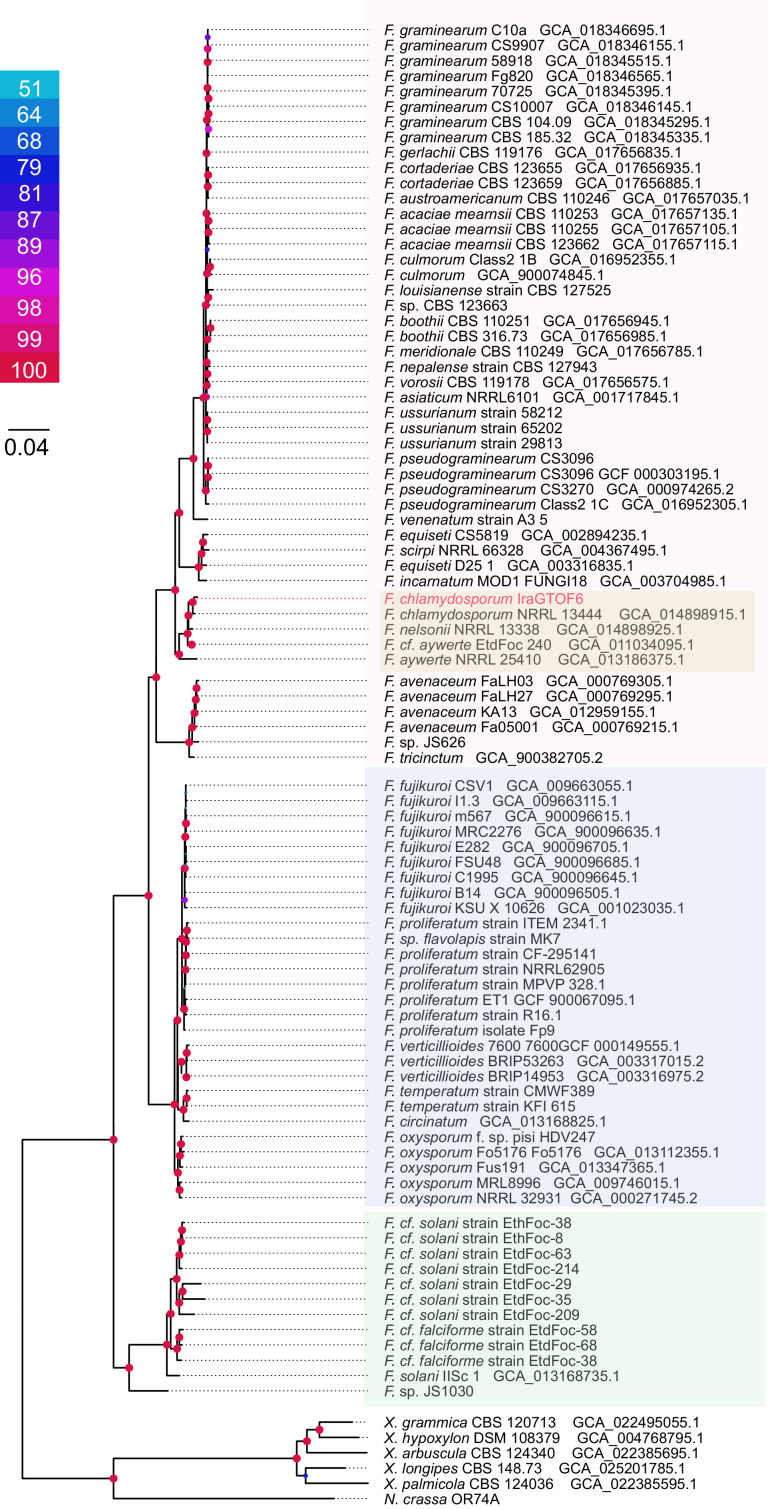
Phylogenomics analysis of *Fusarium* based on proteome core placed IraGTOF6 next to *F. Chlamydosporum* species. Bootstrap values are shown in colored circles, corresponding to the scale in the legend.

To gain a comprehensive understanding of the phylogenetic distribution of *Fusarium* species sequenced at the whole-genome level to date, we downloaded 88 genomes from the genus *Fusarium*, along with five genomes from *Xylaria* and one from *Neurospora crassa* to be used as outgroups for reconstructing the phylogenomics of the *Fusarium* genus. The tree included 94 genomes and was constructed with 1156 groups of orthologs (Fig. 3).

In this tree, IraGTOF6 is positioned within the yellow subclade of the *F. chlamydosporum* complex that comprises three species. The genome most closely related to IraGTOF6 is the only one previously available from *F. chlamydosporum* species strain NRRL 13,444. The closest relatives are F. *nelsonii* and then *F. aywerte* EtdFoc 240. The other available *F. aywerte* genome, strain NRRL 25,410, is slightly distanced from the main clade. It is observed that *F. chlamydosporum* complex is more closely related to *F. graminearum* and *F. pseudograminearum* than to *F. oxysporum*.

## 4 Experimental Design, Materials and Methods

### 4.1 Strains isolation

The soil samples were collected from rhizosphere in a blackberry field in the region of Los Reyes, Michoacán, Mexico. To isolate *Fusarium* strains, 1 g of soil was weighed and mixed with 10 ml of sterile distilled water. The suspension was vortexed at maximum speed for 5 min to ensure thorough resuspension of the sample. Once resuspended, 1 ml of the suspension was taken to prepare three serial dilutions: 1/100, 1/1000, and 1/10,000. From the original sample and its respective dilutions, 100 microliters were plated on a selective medium for *Fusarium.* Three replicates were plated for each concentration, resulting in a total of 12 plates. The selective medium for *Fusarium* consisted of 0.2 g/L of MgSO₄·7H₂O, 0.9 g/L of K₂HPO₄, 0.15 g/L of KCl, 1 g/L of NH₄NO₃, 3 g/L of glucose, 0.2 g/L of pentachloronitrobenzene (Terraclor 75 %), 20 g/L of agar, and 0.25 g/L of chloramphenicol. The plates were incubated for 4 days at 28 °C to allow adequate colony growth. After incubation, colonies were verified, and a block of agar containing a colony was cut and transferred to a new plate with the selective medium. This plate was incubated for 48 h at 28 °C. Finally, a block from the edge of the grown colony on this plate was transferred to PDA medium for final growth and incubated at 28 °C for 3 days to obtain conidia. Strain IraGTOF6 was isolated from single colonies on agar plates of the same medium.

### 4.2 Taxonomic assignment with ITS and β-tubulin

Genomic DNA was extracted using the Quick-DNA Fungal/Bacterial Microprep Kit (Zymo Research Corporation, Irvine, CA, USA) following the manufacturer's instructions. The quality of the extracted DNA was verified through agarose gel electrophoresis and quantified using the Qubit 1X dsDNA High Sensitivity Kit (Invitrogen, Life Technologies, CA, USA).

The ITS and β-tubulin regions were amplified by PCR and sequenced using the Applied Biosystems 3730xl DNA Analyzer, a Sanger sequencing technology system, to determine the strain's species. For phylogenetic analysis, representative ITS and β-tubulin sequences previously reported in the NCBI database were downloaded for comparison. All sequences were aligned using MUSCLE in MEGA11 and manually trimmed. The resulting alignment file was then used to construct phylogenetic trees with default parameters, employing the Neighbor-Joining method and 1000 bootstrap replicates to assess branch support [5].

### 4.3 Whole genome sequencing

Whole-genome sequencing (WGS) was done using short-read and long-read sequencing technologies. Short-read sequencing was outsourced to the QB3-UC Berkeley Genomics Illumina Platform Library Preparation and Sequencing service. Briefly, library preparation involved DNA shearing using Covaris. Stub Y-adapters were ligated to the DNA fragments and extended to full-length indexed adapters through of PCR. Sequencing was performed on an Illumina NovaSeq 6000 platform, generating paired-end reads of 150 bp. The Rapid Sequencing DNA V14 protocol, provided by Oxford Nanopore, was followed to the letter for long-read sequencing. Extracted DNA was barcoded with the Rapid Barcoding Kit V14, pooled, cleaned with AMPure XP Beads, and mixed with a diluted Rapid Adapter. Flow cells R10.4.1 were primed with Flow Cell Flush, Bovine Serum Albumin, and Flow Cell Tether. After mixing with the Sequencing Buffer and Library Beads, the DNA library was loaded to the flow cell of an Oxford Nanopore MinION Mk1B sequencing device. MinKNOW (v3.6.5) was used to control sequencing while basecalling was performed using Guppy (v3.2.10) in high-accuracy mode.

### 4.4 Genome assembly and quality assessment

TrimGalore 0.6.10 [6] was employed to remove adapters and low-quality sequences from the Illumina NovaSeq reads, with those shorter than 40 bp discarded. In the case of Oxford Nanopore reads, the adapter sequences were removed using Porechop 0.2.4 [7], and any reads shorter than 40 bp were deleted using Chopper 0.9.0 [8]. The genome assembly was performed using MaSuRCA 4.1.1 [9] with default parameters, which polished the long reads using the short reads prior to assembly. Duplicate contigs (haplotigs) were removed with purge_dups 1.2.6 [10]. Low complexity and tandem regions were identified with tantan 49 [11], whereas transposable element detection was performed using RepeatModeler 2.0.6 [12]. We compared the assembly quality and completeness of *F. chlamydosporum* IraGTOF6 and NRRL 13,444 with BUSCO 5.6.1 [13] using the Hypocreales ODB10 dataset as reference.

### 4.5 Genome annotation

The resulting masked genome was used as input for structural and functional annotation with Funannotate 1.8.17 [14], employing the pre-trained gene finding weights from Augustus 3.5.0 [15] for *F. graminearum* and mapping the sequences to 400,000 gene models from UniProt [16]. The functional annotation was further extended with predictions from InterProScan 5.59_91.0 [17], antiSMASH 6.1.1 [18], and EggNOG mapper 2.1.12 [19].

### 4.6 Phylogenomics analysis

To obtain the species tree in Fig. 3, we calculated our dataset's core proteome of the 94 genomes. The core was calculated using usearch v10.0.240 to calculate orthologous protein clusters [20] as implemented in BPGA v1.3 [21]. The core proteome consisted of 1156 proteins, which were aligned, trimmed, and concatenated to construct a partitioned matrix. The matrix was then used for phylogenetic analysis with IQtree v 2.0.7 [22]; a model was selected for each partition using modelFinder implemented in IQtree [23]. The branch support was calculated using ultrafast bootstrapping for 10,000 replicates. To obtain phylogenomic trees, BPGA outputs were processed using an automated pipeline available at github.com/WeMakeMolecules/Core-to-Tree/tree/v1.02.

## Limitations

Not applicable.

## Ethics Statement

All authors have reviewed and adhered to the ethical guidelines required for publication in Data in Brief. They confirm that this work does not involve animal experiments, or the use of data obtained from social media platforms.

## Credit Author Statement

**Anton Pashkov:** Writing- Original Draft, Data Curation, Formal analysis, Investigation; **José Pedro Martínez-Hernández:** Methodology, Writing - Review & Editing; **Alfredo Herrera-Estrella:** Methodology, Writing - Review & Editing; **Pablo Cruz-Morales:** Investigation, Data Curation; **Nelly Selem-Mojica:** Funding acquisition, Supervision, Conceptualization. Writing - Review & Editing; **José Manuel Villalobos-Escobedo:** Funding acquisition, Supervision, Conceptualization. Writing - Review & Editing.

## Data Availability

NCBISRX27535412: Illumina WGS of Fusarium chlamydosporum: causal agent of Fusarium wilt in strawberry field (Original data). NCBI/SRAFusarium chlamydosporum genome sequencing, assembly, and annotation (Original data). ZenodoData for ``Hybrid de novo genome assembly and comparative genomics of Fusarium chlamydosporum isolated from infected strawberry fields'' (Original data). NCBISRX27535413: Oxford Nanopore WGS of Fusarium chlamydosporum: causal agent of Fusarium wilt in strawberry field (Original data).
